# Proselfs depend more on model-based than model-free learning in a non-social probabilistic state-transition task

**DOI:** 10.1038/s41598-023-27609-0

**Published:** 2023-01-25

**Authors:** Mineki Oguchi, Yang Li, Yoshie Matsumoto, Toko Kiyonari, Kazuhiko Yamamoto, Shigeki Sugiura, Masamichi Sakagami

**Affiliations:** 1 Brain Science Institute, Tamagawa University, 6-1-1, Tamagawagakuen, Machida, Tokyo, Japan; 2 Graduate School of Informatics, Nagoya University, Nagoya, Japan; 3 Department of Psychology, Faculty of Human Sciences, Seinan Gakuin University, Fukuoka, Japan; 4 School of Social Informatics, Aoyama Gakuin University, Kanagawa, Japan; 5 Genesis Research Institute, Aichi, Japan

**Keywords:** Psychology, Human behaviour

## Abstract

Humans form complex societies in which we routinely engage in social decision-making regarding the allocation of resources among ourselves and others. One dimension that characterizes social decision-making in particular is whether to prioritize self-interest or respect for others—proself or prosocial. What causes this individual difference in social value orientation? Recent developments in the social dual-process theory argue that social decision-making is characterized by its underlying domain-general learning systems: the model-free and model-based systems. In line with this “learning” approach, we propose and experimentally test the hypothesis that differences in social preferences stem from which learning system is dominant in an individual. Here, we used a non-social state transition task that allowed us to assess the balance between model-free/model-based learning and investigate its relation to the social value orientations. The results showed that proselfs depended more on model-based learning, whereas prosocials depended more on model-free learning. Reward amount and reaction time analyses showed that proselfs learned the task structure earlier in the session than prosocials, reflecting their difference in model-based/model-free learning dependence. These findings support the learning hypothesis on what makes differences in social preferences and have implications for understanding the mechanisms of prosocial behavior.

## Introduction

In this study, we investigated how domain-general learning mechanisms relate to people's social preferences in decision-making. Several studies have supported the idea that humans and many other animals have at least two mechanisms for controlling their behavior based on their experiences in the environment^1–10^. One is to prospectively infer the contingency of actions and outcomes while taking into account the probabilistic transition relations between events in the environment, and the other is to retrospectively link actions and rewards without considering the structure of the environment. These mechanisms have been computationally formulated in the framework of reinforcement learning^1–4^. The former “model-based” system builds an internal model of the environment and selects actions that maximize rewards by learning state-action values while estimating state-transition probabilities. The latter “model-free” system reinforces and repeats the behavior that results in the reward but does not learn the contingencies between states in the environment. The model-based system supports goal-directed behavior and is flexible to changes in the environment; however, the processing is sequential and computationally expensive. The model-free system supports habitual behavior, has a low computational cost, and works automatically once learning is established.

These systems are of contrasting natures and can make decisions that are incompatible with each other. Nevertheless, it seems that the two systems are not mutually exclusive and coexist in our brains to support the optimization of our behavioral strategies. In line with this view, in behavioral tasks to study the model-based/model-free systems, a hybrid model of the two fits the behavioral data better than either alone^3,4,11,12^. In addition, the mixing ratio of model-based and model-free can vary between and within individuals. This variability in mixing weight can contribute to balancing reward maximization and cognitive resource allocation in learning^3,13,14^. For example, in a novel task, the model-based system may dominate the early phase of learning and arrive at the best option faster by learning the structure of the environment. The model-free system then becomes dominant in the later phase of learning, making the optimal behavior habitual and reducing behavioral costs^15^. Arbitration between model-based and model-free strategies would be achieved either by mixing the two, or by switching between them^2,13,14,16,17^. In this study, we adopted the former framework to examine changes in the mixing weights among and within individuals.

The model-based/model-free systems have been studied mainly in the context of individualistic economic decision-making involving the maximization of rewards such as food and money. However, we humans do not live in isolation, but form complex societies with other conspecifics. We routinely exchange tangible and intangible resources with others in our daily lives. These social decisions on resource allocation are influenced by the preference that pursues the maximization of self-interest without considering others (proself) and the preference that respects others’ interests and avoids unequal distribution (prosocial). Researchers have defined these social preferences as social value orientation (SVO)^18–20^. SVO is a stable personal trait regarding how people evaluate self-prioritizing and other-respecting decisions in the allocation of resources in social settings.

What accounts for individual differences in social preferences? On the one hand, some theorists emphasize that we have domain-specific functional modules for social decision-making because of the importance of social exchange in our adaptation, and that in this respect social decision-making is unique and goes beyond the general framework of decision-making^21–23^. These theorists would argue that individual differences in social preferences stem from the differences in the balance of social module functions. However, on the other hand, there is a view that resource allocation in social decision-making is not special and shares much of the framework with the usual economic decision-making processes^24,25^. Recent developments in the dual-process theory in social decision-making are in line with the latter view. The conventional dual-process theory^23,26–28^ argues that our moral or social decision-makings are processed by two systems working in parallel (so-called “System 1” and “System 2”) and that System 1 and 2 have contrasting properties such as intuition vs. deliberation, automaticity vs. control, and emotion vs. cognition. In recent years, some theorists have begun to reconsider the social dual-process theory from the perspective of learning mechanisms^25,29–31^. According to this idea, the difference between Systems 1 and 2 in the social dual-process theory should be attributed to the duality of the underlying domain-general learning mechanism (i.e., the model-free/model-based systems). This “learning” approach, although still only a theoretical proposal and lacking empirical basis, leads to the hypothesis that individual differences in social preferences are due to the variability in the balance of the model-free/model-based systems. An experimental test of this hypothesis provides empirical support for the learning approach in the social dual-process theory.

To achieve this goal, we measured SVO in participants who performed a sequential two-choice Markov decision task, which allowed us to differentiate between the effects of model-free and model-based learning. In this task, the reward contingency is fixed for each state of the final choice, which enabled us to examine the change in the weight for model-based and model-free learning for an individual. The results showed that proselfs had a larger mean reward gain in the early phase of the session than prosocials, and that the difference in reaction time due to learning different transition probabilities between states occurred earlier in proselfs than in prosocials. Parameter estimation using reinforcement learning models showed that proselfs had greater model-based dependence than prosocials in the early phase of the session. In the later phase, model-based dependence gradually decreased, and these differences between proselfs and prosocials became smaller. These analyses indicate that proselfs learned the task structure through model-based learning, and thus arrived at the optimal options more quickly than prosocials. This study suggests that the inter-individual variability in SVO is due to individual differences in model-free/model-based dependence and that social decision-making uses domain-general learning systems.

## Results

### Sequential two-choice Markov decision task

**Figure 1:**
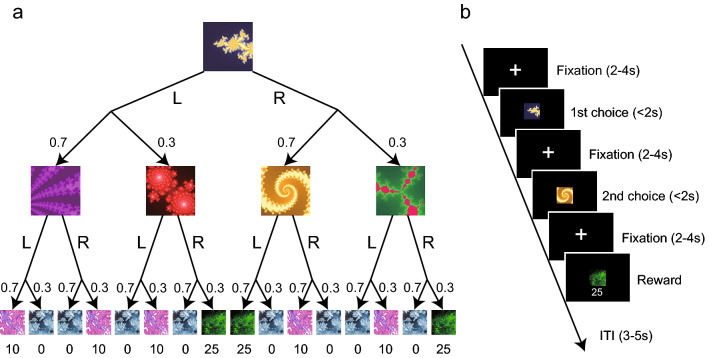
Sequential two-choice Markov decision task. (**a**) The probability transition structure of the task. Each state at the first choice, second choice, and outcome is composed of a geometric figure. The first stage starts with a common figure. The participant chooses left or right, and probabilistically (70%/30%) transitions to the corresponding next figure. In the second stage, the participant again chooses left or right, and finally arrives at a figure that is associated with a different monetary reward (0, 10, or 25 yen). (**b**) Time sequence of the task. First, the fixation point is presented, and then the figure of the first stage is presented. The participant makes a first choice within 2 s, and after the fixation point is presented again, the participant moves to the second stage. When the figure of the second stage is presented, the participant makes a second choice within 2 s. After the presentation of the fixation point, the reward information is given, and after the inter trial interval, the next trial starts.

In this study, the participants were asked to perform a sequential two-choice Markov decision task^4^ online to investigate model-based and model-free dependency in learning the task structure (Fig. 1a). In this task, each participant made a choice in each of the two consecutive stages, leading to an outcome state that was associated with a specific amount of reward (Fig. 1b). Each state-action led to a probabilistic transition to one of the two branching states. The first stage consisted of a fixed geometric figure, in which the participant selected either left or right using a keypress within 2 s. After the choice, the participant moved to one state with a probability of 70% (common transition) and to another state with a probability of 30% (rare transition). Each of the four states that constituted the second stage corresponded to a different geometric figure. Each participant also had to choose either left or right within 2 s in the second stage. The participant then moved probabilistically (70% vs. 30%) to one of the two outcome states depending on the second choice. The third stage was associated with a specific reward amount (0, 10, or 25 yen), each of which was associated with the same geometric figure. In the first stage, the right choice had the largest state-action value (14.5). In the second stage, the left choice in the state moved by a common transition after the right choice in the first stage had the largest state-action value (17.5). The transition probabilities between states were fixed throughout the session. Participants performed 200 trials of this task, and the total reward gain was added to the baseline participation fee.

### The SVO scores and its distribution

**Figure 2:**
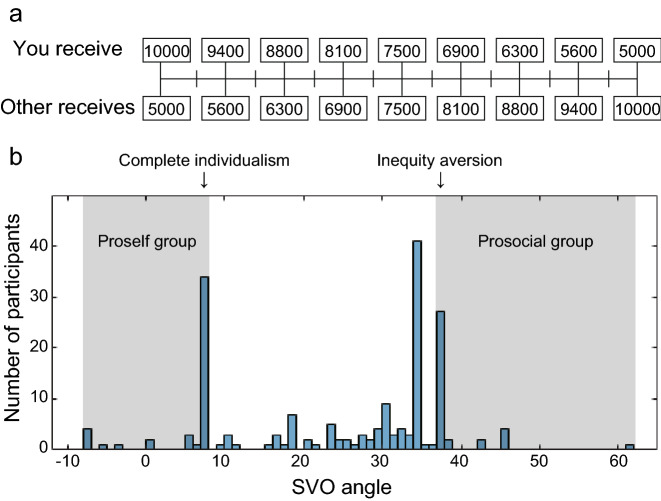
Measurement and distribution of SVO values. (**a**) SVO slider measure. The participant choose one of nine different allocation patterns of the amount he or she receive and the amount received by the other. (**b**) The histogram of SVO scores. In this study, the prosocial group was classified according to the score of inequity aversion or higher, and the proself group according to the score of complete individualism or lower (light gray areas).

A total of 184 participants (92 females, 1 no-answer) participated in this experiment. One participant was excluded from the analysis because of missing personal and SVO data. After performing the decision-making task, the SVO scores of the participants were measured using the SVO Slider Measure^20^. This measurement quantifies SVO as an angle in a two-dimensional space with the axis of self- and other-payoffs by analyzing choices regarding resource allocation between the self and others (Fig. 2a). In the study that devised this measurement^20^, the angle from 57.15° to 22.45° was defined as prosocial, 22.45° to 12.04° as individualist, an angle larger than 57.15° as an altruist who favors others over him or herself, and an angle smaller than 12.04° as a competitor who tries to maximize the difference between the self and others. The SVO scores in our participant group ranged from a minimum of -7.19° to a maximum of 61.39° (Fig. 2b). Previous studies have shown that participants with higher (or lower) SVO scores exhibit greater cooperative (or selfish) behavior^32,33^, and therefore, such participant groups may have distinctive characteristics in decision-making in general. To focus on participants with pronounced prosocial and proself tendencies, we classified participants who scored above the typical prosocial choice of inequity aversion (37.48°) as the prosocial group (n = 35, 19.1%) and those who scored below the typical proself choice of complete individualism (7.82°) as the proself group (n = 46, 25.1%) and analyzed how they differed in behavioral measures and learning model estimates. The same statistical comparisons were applied to the prosocial and proself groups classified by the conventional criterion of more or less than 22.45° (Prosocial: n = 117, 63.9%; Proself: n = 66, 36.1%) as complementary analysis. Generalized linear mixed-effects models (GLME) were used to examine the effects of gender and age on SVO scores separately, controlling for the other as a random effect. Neither gender nor age had a significant effect on SVO (Gender: *Estimate* = 1.00, *SE* = 0.65, *t-value* = 1.54, *p* = 0.126; Age: *Estimate* = −0.20, *SE* = 0.20, *t-value* = -0.99, *p* = 0.322).

### The difference in reward gain between the proself and prosocial groups

**Figure 3:**
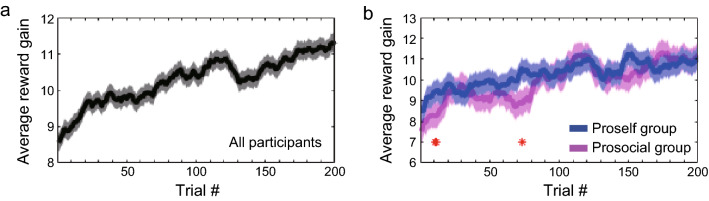
Comparing reward gain between the proself and prosocial groups. (**a**) Moving averages of the reward amount for all participants, where shaded error bar represents standard error of the mean (SEM). (**b**) Moving averages of the reward amount for the proself and prosocial groups; Blue indicates the proself group. Magenta indicates the prosocial group. Asterisks indicate data points where there was a significant difference (*p* < 0.05) between the proself and prosocial groups. Significant differences were found in trials #11, #12, and #73.

We first analyzed the moving average of reward amounts with a 20-trial range in one trial step for all participants. We found that the average reward amount tended to increase gradually as the session progressed (Fig. 3a). This indicates that participants generally learned through experience to make choices with higher expected values in each state. We then calculated the average reward amounts separately for the proself and prosocial groups and compared their performances (Fig. 3b). The results showed that, especially in the early phase of the session (around the first 20 trials, and 60–80 trials), the mean reward amount was significantly larger in the proself group than in the prosocial group. As the session progressed, the difference between the two groups disappeared. The total reward amounts were larger for the proself group, but not significant (Prosocial: 1993.1 yen ± 413.1 SD, Proself: 2049.8 yen ± 378.5 SD, *t*[79]  = 0.64, *p* = 0.523, two-sample *t*-test). When the participants were divided by SVO angle 22.45°, no significant differences between groups were found in either timing (Supplementary Fig. 1).

### The difference in reaction times between the proself and prosocial groups

**Figure 4:**
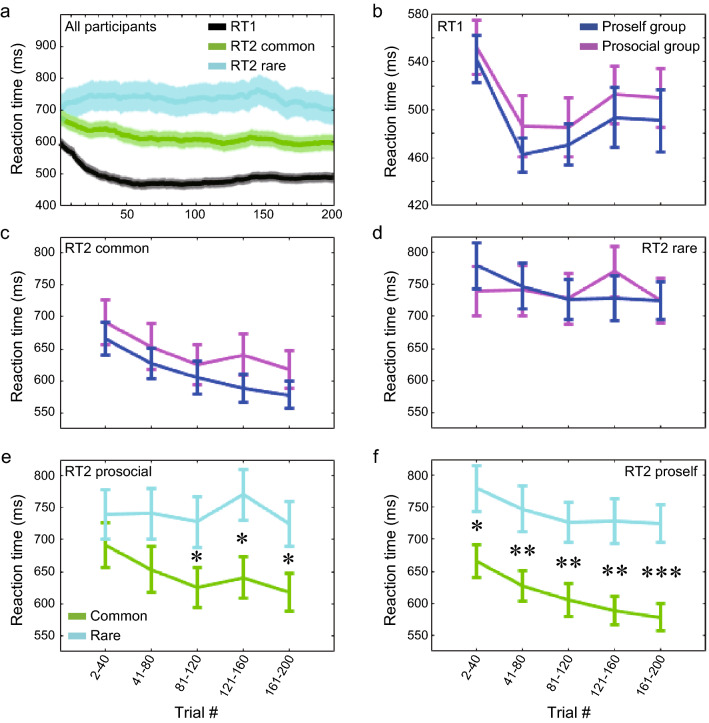
Comparing reaction times between the proself and prosocial groups. (**a**) Reaction times averaged across all participants for the first choice (RT1: black), the second choice after common transitions (RT2 common: green), and the second choice after rare transitions (RT2 rare: cyan). Shaded error bar represents SEM. (**b**) Averaged reaction times per 40 trials at the first choice, divided into the proself and prosocial groups. Blue indicates the proself group. Magenta indicates the prosocial group. Error bar indicates SEM. (**c**) RTs at the second choice after common transitions, (**d**) RTs at the second choice after rare transitions, (**e**) RTs at the second choice of the prosocial group divided into after common and rare transitions. Green represents after common transitions, and cyan represents after rare transitions. (**f**) RTs at the second choice of the proself group. *p < 0.05, **p < 0.01, ***p < 0.001.

Next, we analyzed the reaction time (from stimulus presentation to key-press). First, we calculated the trial-by-trial average of reaction time in the first choice (RT1) and in the second choice (RT2) for all participants (Fig. 4a). In the second choice, we separated the reaction times after the common and rare transitions from the first state-action. RT1 and RT2 after common transitions decreased after the start of the session, while RT2 after rare transitions increased. The first state was always the same and could be predicted decisively, whereas the second state was not. In addition, as learning about the task structure progressed, participants anticipated common transitions and, thus, prepared to respond to the state after them. Consistent with these task characteristics, in the stable state during the second half of the session, RT1 was the shortest, followed by RT2 after the common transition, and RT2 after the rare transition was the longest (averaged over trial #101–200; RT1: 484.5 ms, RT2 after the common transition: 603.6 ms, RT2 after the rare transition: 732.8 ms).

Next, we analyzed the average reaction time in blocks of 40 trials each, dividing the participants into proself and prosocial groups. For RT1, a two-way repeated measures analysis of variance (ANOVA) with social preference (proself vs. prosocial) and block (blocks 1–5) as factors showed a main effect of block (Fig. 4b; *F*[4,82] = 10.93, *p* = 2.47 × 10^–8^). RT1 was the longest at the beginning of the session and then decreased. Table 1 shows the results of GLME for RT1 with trial number and social preference as fixed effects while controlling for participant ID, gender, and age as random effects. The effect of trial number was significant. In RT2 after common transition, there was a main effect of block (Fig. 4c; *F*[4,79] = 8.46, *p* = 1.71 × 10^–6^, a two-way repeated measures ANOVA). In RT2 after rare transition, there was no significant main or interaction effect (Fig. 4d). Next, we compared RT2 after common and rare transitions for each group. In the prosocial group, a two-way repeated measures ANOVA, with transition probability (common vs. rare) and block (blocks 1–5) as factors, showed main effects of transition probability (Fig. 4e; *F*[1, 34]  = 27.49, *p* = 8.30 × 10^–6^) and block (*F*[9, 34]  = 7.12, *p* = 2.25 × 10^–9^) and their interaction effect (*F*[1, 34]  = 25.14, *p* = 1.65 × 10^–5^). In the proself group, a two-way repeated measures ANOVA showed main effects of transition probability (Fig. 4f; *F*[1, 45]  = 65.22, *p* = 2.67 × 10^–10^) and block (*F*[9, 45]  = 17.52, *p* = 1.58 × 10^–24^) and their interaction effect (*F*[1, 45]  = 58.64, *p* = 1.09 × 10^–9^). Table 1 also shows the results of GLME for RT2 with trial number, SVO group, transition as fixed effects. The effect of transition and all interactions of two factors were significant. Comparing RT2 after common and rare transitions block by block, there were significant differences from the first to the end blocks for the proself group (Block1: *t*[90] = 2.56, *p* = 0.012, Block2: *t*[90] = 2.77, *p* = 0.007, Block3: *t*[90] = 3.02, *p* = 0.003, Block4: *t*[90] = 3.36, *p* = 0.001, Block5: *t*[90] = 4.09, *p* = 9.44 × 10^–5^, two-sample *t*-test), while, in the prosocial group, there was no significant difference in the first and second blocks, and there were significant differences from the third block onward (Block1: *t*[68] = 0.92, *p* = 0.357, Block2: *t*[68] = 1.64, *p* = 0.107, Block3: *t*[68] = 2.03, *p* = 0.047, Block4: *t*[68] = 2.51, *p* = 0.015, Block5: *t*[68] = 2.34, *p* = 0.022). This result suggests that the proself group learned the transition probabilities between states earlier in the session than the prosocial group. When the participants were divided by SVO angle 22.45°, there were significant differences in RT2 after common and rare transitions from the first block for both groups (Supplementary Fig. 2a–d).

**Table 1:** Generalized linear mixed-effects models for the reaction times (RT1 and 2).

	Fixed effect	Estimate	SE	t-value	p
RT1	Intercept	535.33	25.05	21.38	5.51 × 10^–100^
	Trial	-0.24	0.04	-5.96	2.65 × 10^–9^
	SVO	-14.32	27.1	-0.53	0.597
	Trial*SVO	-0.05	0.05	-0.86	0.388
RT2	Intercept	735.73	34.96	21.05	5.08 × 10^–97^
	Trial	0.04	0.10	0.47	0.641
	SVO	25.32	38.48	0.66	0.510
	Transition	-43.32	13.3	-3.26	1.12 × 10^–3^
	Trial*SVO	-0.30	0.13	-2.36	0.018
	Trial*transition	-0.47	0.11	-4.14	3.46 × 10^–5^
	SVO*transition	-56.59	17.65	-3.21	1.35 × 10^–3^
	Trial*SVO*transition	0.18	0.15	1.16	0.247

### The difference in estimated model parameters between the proself and prosocial groups

To investigate the differences in decision-making mechanisms between the proself and prosocial groups, we estimated the model parameters for each group using reinforcement learning models with the behavioral data. As reinforcement learning models, we used the model-free SARSA model, model-based FORWARD model, and HYBRID model that combined them, as used in a previous study^4^. The SARSA model uses temporal difference (TD) errors to calculate state-action values, and only considers information on reward prediction errors, not the structure of the environment. In contrast, the FORWARD model uses state prediction errors to learn the structure of the environment (transition probabilities between states according to actions) and combines them with reward prediction errors to calculate the state-action values. Previous studies have shown that participants’ behavior can be best fitted by the HYBRID model that combines the state-action values computed from model-free and model-based learners with a certain weight *w*^3,4^. We divided the participants’ behavior into blocks of 40 trials each and conducted individual-level analysis using the maximum a posterior (MAP) estimation to estimate the free parameters in each block for each participant (see the “Methods” section). We used four free parameters: the learning rate *α* for the model-free learner and *η* for the model-based learner, which are related to how strongly the association strength is updated per trial; an inverse temperature *β* that affects the probability of taking particular actions based on the relevant state-action values (the smaller β, the more exploratory the action will be under the same values); and weight *w*, which characterizes the balance between model-based and model-free learning (the larger the *w*, the greater the dependence on model-based learning). We calculated the Bayesian information criterion (BIC) for each model using data from all participants, and the HYBRID model showed the best goodness-of-fit for all blocks (Supplementary Fig. 3).

**Figure 5:**
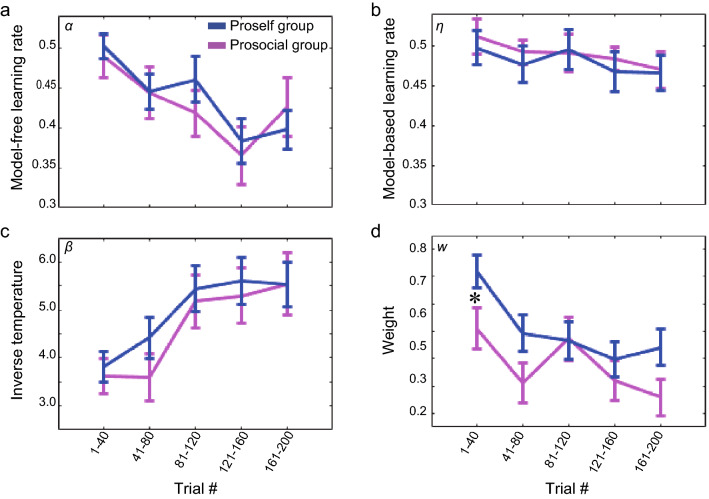
Comparing estimated parameters between the proself and prosocial groups. (**a**) Model-free learning rate (*α*) estimated by the model analysis, divided into the proself and prosocial groups. Blue indicates the proself group; magenta indicates the prosocial group. Error bar indicates SEM. (**b**) Model-based learning rate (*η*). (**c**) Inverse temperature (*β*), (**d**) weight (*w*) representing model-based dependence. *p < 0.05.

Next, we estimated the free parameters separately for the proself and prosocial groups using the HYBRID model and compared these estimates. A two-way repeated measures ANOVA for model-free learning rate *α* (Fig. 5a), with social preference and block as factors, showed a main effect of block (*F*[4,82] = 5.49, *p* = 2.75 × 10^–4^). A GLME for *α* with block and social preference as fixed effects also showed that the effect of block was significant (Table 2). Learning rate *α* was largest in the first block and then decreased. There was no significant difference in model-based learning rate *η* in either comparison (Fig. 5b and Table 2). For inverse temperature *β* (Fig. 5c), the main effect of block was found (*F*[4,82] = 9.76, *p* = 1.80 × 10^–7^). A GLME for *β* also showed that the effect of block was significant (Table 2). Inverse temperature *β* was the smallest in the first block and then increased. For weight *w* (Fig. 5d), the main effects were found for both social preference (*F*[1,82] = 8.96, *p* = 0.0037) and block (*F*[4,82] = 5.01, *p* = 6.26 × 10^–4^). A GLME for *w* showed that the effect of block was significant, while the effect of social preference was marginal (Table 2). Weight *w* was large in the first block and then tended to decrease. The proself group generally had a larger weight than the prosocial group. No interaction was observed in any of the above comparisons for the model parameters. However, behavioral analysis of reward gain and reaction time showed that there were differences between the proself and prosocial groups, especially in the initial block. This suggests that the lack of interaction might be due to the fact that the learning that led to differences between the proself and prosocial groups was done at an early phase in the session, and there were many remaining trials after that. Therefore, we conducted a two-way repeated-measures ANOVA with the model parameters using the data from the first 80 trials. We used 80 trials here because that was the total number of trials in a previous study that used the same task^4^. The results showed an interaction between social preference and block only for weight w (*F*[1,79] = 7.37, *p* = 0.008). A post-hoc analysis showed that, when the proself and prosocial groups were compared block-by-block, a significant difference was found only in the first block (*z* = 2.19, *p* = 0.037). This indicates that the proself group was more model-based dependent in the early phase of the session than the prosocial group. When the participants were divided by SVO angle 22.45°, there were no significant differences between groups for *α*, *η*, *β* and *w* in any of the blocks (Supplementary Fig. 4a–d).

**Table 2:** Generalized linear mixed-effects models for parameter estimates.

	Fixed effect	Estimate	SE	t-value	p
*α*	Intercept	0.51	0.03	16.28	3.99 × 10^–46^
	Block	-0.02	9.48 × 10^–3^	-2.63	8.75 × 10^–3^
	SVO	-2.73 × 10^–4^	1.18 × 10^–3^	-0.23	0.817
	Block*SVO	3.79 × 10^–5^	3.56 × 10^–4^	0.11	0.915
*η*	Intercept	0.50	0.03	20.03	2.47 × 10^–62^
	Block	-8.12 × 10^–3^	7.37 × 10^–3^	-1.10	0.271
	SVO	3.68 × 10^–4^	9.37 × 10^–4^	0.39	0.695
	Block*SVO	4.27 × 10^–6^	2.76 × 10^–4^	0.02	0.988
*β*	Intercept	3.57	0.53	6.73	5.77 × 10^–11^
	Block	0.46	0.13	3.59	3.66 × 10^–4^
	SVO	-0.01	0.02	-0.63	0.532
	Block*SVO	1.73 × 10^–3^	4.87 × 10^–3^	0.35	0.723
*w*	Intercept	0.73	0.08	9.26	1.29 × 10^–18^
	Block	-0.07	0.02	-2.80	5.28 × 10^–3^
	SVO	-5.23 × 10^–3^	2.93*10^–3^	-1.78	0.075
	Block*SVO	3.96 × 10^–4^	8.84*10^–4^	0.45	0.654

Thus far, we have used individual-level analysis; however, if the number of trials used for fitting is small, we need to be cautious about its reliability. Therefore, to confirm the validity of the results of the individual-level analysis, we conducted a fixed-effects analysis in which the parameters were estimated to be identical for each group member (Supplementary Fig. 5a–d). The results showed similar patterns to the individual-level analysis for all parameters. It is worth noting that a large difference between the proself and prosocial groups was observed in the first block in the estimates of weight *w* as well. In addition, to confirm that the parameters estimated in the individual-level analysis were legitimate, a hybrid model was used to fit the 200 trials without dividing them into blocks. All estimates were within the range of the values calculated for the blocks (Proself: α = 0.34 ± 0.04, η = 0.49 ± 0.03, β = 5.21 ± 0.48, w = 0.49 ± 0.05; Prosocial: α = 0.33 ± 0.04, η = 0.48 ± 0.03, β = 4.58 ± 0.55, w = 0.40 ± 0.06). The correlation matrices of the parameters estimated for each block using individual-level analysis are shown in the Supplementary Information (Supplementary Fig. 6). No correlations between parameters were found in the first block; however, some parameters were correlated in later blocks.

### Simulating model-based and model-free learners

**Figure 6:**
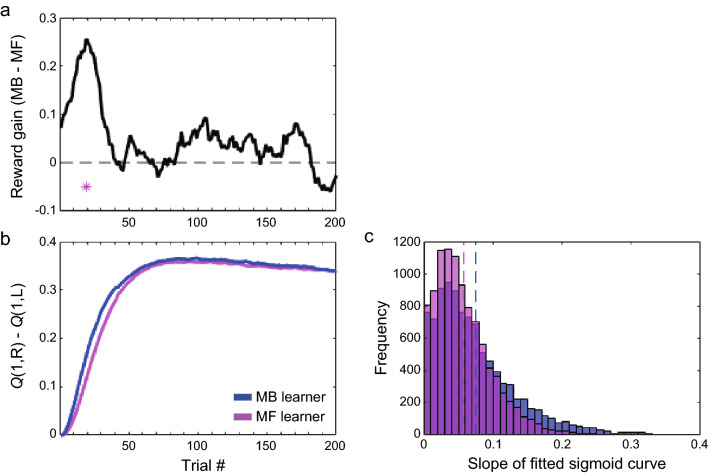
Simulating model-based and model-free behavior. (**a**) Difference between rewards earned by the model-based and model-free learners simulated with different weight parameters of the HYBRID model. * indicates *p* < 0.001. (**b**) State-action values at the first choice (*Q*[1,R] for the right choice, *Q*[1,L] for the left choice) estimated from simulations of model-free (magenta) and model-based (blue) learners. (**c**) The histogram of the slope of the sigmoid function fitted to the difference of state action values in (**b**). The dotted lines represent the mean.

In the behavioral analysis, the proself group had a larger average reward gain in the early phase of the session than the prosocial group, and there was already a significant difference in the reaction times after common and rare transitions in the early phase in the proself group, but not in the prosocial group. The proself group was also more model-based dependent early in the session than the prosocial group. We simulated model-based and model-free learners to confirm the consistency with these results. We created a model-based learner by setting the weight *w* of the HYBRID model to 1 in the early phase of learning (1–40 trials) and a model-free learner by setting it to 0. The weights of the remaining blocks were all set to 0.5. For the other free parameters, we used the estimates obtained from the fixed-effects analysis of a previous study^4^. We ran 10,000 iterations for each of the model-free and model-based simulations, and first calculated the moving average of the reward gain from the output data. Consequently, the reward obtained by the model-based learner was larger than that obtained by the model-free learner in approximately 20 trials (Fig. 6a). Next, to examine the speed of learning, we computed the difference between the estimated state-action values for the left and right choices at the first choice, *Q*(1,R)–*Q*(1,L). For the first choice, the right choice has a larger state-action value than the left choice at the ground truth; thus, *Q*(1,R)–*Q*(1,L) should increase from the initial value of 0 to a positive value as learning progresses. As a result, *Q*(1,R)–*Q*(1,L) gradually increased in the early phase of the session for both model-based and model-free simulations, but the values were consistently larger for the model-based than model-free (Fig. 6b). To compare the learning speed, we fitted *Q*(1,R)–*Q*(1,L) in individual repetitions with a sigmoid function transformed to pass through the origin to obtain the value of the slope. The slope values were larger for the model-based than for the model-free (Fig. 6c; *z* = 17.02, *p* = 6.45 × 10^–65^, Wilcoxon rank-sum test), indicating that the model-based learner has a greater learning speed in the early phase of the session.

Finally, to confirm that simulations with parameters estimated from behavioral data recover behavioral outcomes, we made simulations with the HYBRID model using parameters estimated from behavioral data of the proself and prosocial groups, and compared reward gains and the learning speeds of state-action values. Here, we used estimates from a fixed effects analysis because it was more reliable than the individual-level analysis. The results replicated behavioral data showing that the proself actor had greater reward gain and learned state-action values faster than the prosocial actor in the early stage of the session (Supplementary Fig. 7a–c).

## Discussion

To examine the relationship between inter-individual variability in social decision-making and that in domain-general decision-making, this study used a non-social probabilistic state-transition task to compare model-based/model-free dependence between groups classified by their social preferences. The results of the behavioral analysis showed that the average amount of reward gradually increased as the trial progressed, and the proself group gained a greater amount of reward than the prosocial group in the early phase. In the reaction time analysis at the second choice, which reflected learning of transition probabilities, the proself group was able to distinguish between common and rare transitions from the early phase of the session, whereas the prosocial group did not. These results suggest that the proself group learned the structure of the task more quickly than the prosocial group. We then performed parameter estimation using a hybrid model that combined model-free and model-based reinforcement-learning models. Examining the parameters every 40 trials, we found that the learning rate (especially the model-free *α*) was high at the beginning and gradually decreased. The inverse temperature parameter was conversely low at first and gradually increased. These results suggest that the participants learned the task structure in the early phase of the session and then used this knowledge to take stable actions with high reward expectations. The weight parameter was larger for the proself group than for the prosocial group in the initial phase, indicating that the proself group was more model-based dependent, while the prosocial group was more model-free dependent in the early phase of the session where the task structure was intensively learned. In the later phase, the weight estimates of the proself group decreased and there was no longer a significant difference between the proself and prosocial groups. These differences in behavior and model estimates were found only between groups with prominent proself and prosocial tendencies since participants with intermediate SVO scores did not show distinctive behavioral characteristics. The simulation results obtained using computational models were also consistent with the results of the behavioral analysis. The model-based learner learned state-action values more quickly than the model-free learner, and its average rewards were greater in the early phase of the session. These results suggest that the proself group was more model-based dependent in learning the task structure than the prosocial group and therefore learned each state-action value more quickly to reach a more efficient choice set.

The above result, that proselfs were more model-based dependent and prosocials were more model-free dependent, supports the recent development of the dual-process theory in the social domain. Conventionally, Systems 1 and 2 in the social dual-process theory have been characterized by opposing features, such as intuition vs. deliberation, automaticity vs. control, and emotion vs. cognition^23,26–28^. In recent years, however, several theorists have criticized the fact that these opposing features do not properly distinguish between the two systems^25,29–31^. According to these critics, the dual systems are most appropriately distinguished by different learning processes: model-based and model-free learning. This “learning” approach insists that, for example, in a moral dilemma such as the trolley problem, deontological decisions that avoid forcing sacrifices on the few in order to save the many originate from the model-free system, while utilitarian decisions that affirm forcing sacrifices originate from the model-based system^31^. The results in the various versions of the trolley problem can be properly explained by which learning system these choices come from but not by which of the opposing properties described above is at work. The contrasting features traditionally thought to exist between Systems 1 and 2 are only secondary to these differences in the learning process. However, this learning approach in the social dual-process theory is still only a theoretical proposal and has not been directly tested experimentally. The current study provides empirical support for this learning approach by showing that differences in dependence on the two learning systems are associated with differences in social preferences.

If the learning approach in the social dual-process theory is correct, there should be common features and common neural substrates for the mechanisms at work in social and non-social settings. Indeed, while model-free dependence becomes stronger when time pressure, cognitive load, or acute stress increases^17,34,35^, shorter reaction times result in greater emphasis on default social preferences as well^36,37^. The dorsolateral prefrontal cortex (DLPFC) plays a central role in various functions involving model-based processing^4,6,15,38–40^. An fMRI study showed that proselfs had a larger DLPFC volume and stronger activity in the same region in the prisoner's dilemma game^41^. The same study also showed that prosocials, in contrast, had a larger amygdala volume, which is involved in automatic emotional processing, and stronger activity in the same region. The DLPFC is active both in the decision of prosocials to betray and in that of proselfs to cooperate in the prisoner's dilemma game^42^. Amygdala activity predicts inequity-aversion in prosocials^43^. Calculations of value and probability in moral judgments are based on the same neural substrates as those used for calculations in economic judgments, such as the ventral medial prefrontal cortex, ventral striatum, and anterior insula^44^. In addition, some studies have shown that social and monetary rewards are processed by common brain regions, such as the striatum, which is involved in the reward prediction error^45,46^. Unlike these previous studies, the findings of the present study directly provide evidence to encourage the development of the social dual process theory from the learning framework and provide important insights into the mechanisms underlying prosocial behavior and its inter- and intra-personal diversity.

The results of parameter estimation using the HYBRID reinforcement learning model that mixed SARSA and FORWARD revealed that weight *w*, which indicates the strength of model-based dependence, was large for the proself group at the beginning of the session and rapidly decreased as the session progressed. This result is consistent with a previous study, where it was shown that weight was best fitted by exponential decay^4^, although it did not consider social preferences. It is reasonable to assume that the proself group used the cost-demanding model-based system in the early phase, where participants were active in learning the task structure, and then shifted to the less costly automatic model-free system. In support of this, it has been shown that people adaptively shift the balance between model-free and model-based systems through online reliability assessments and cost–benefit analyses^13,14^. The shift in the model-based weight observed in this study may be due to this arbitration mechanism. Besides the view that arbitration is performed by mixing the two strategies, which is adopted in our study, another view exists: arbitration performed by switching between them. In this switch framework, a single model-based or model-free algorithm was chosen in each trial. This had the advantage of reducing the computational cost compared to running two or more algorithms in parallel, while there was some evidence that such parallel processing occurred in the brain^4,13,47^. It will be a matter of future work to determine which one is the appropriate view.

Reaction time analysis at the second choice showed that the proself group was able to distinguish between common and rare transitions earlier than the prosocial group. Additionally, the average reward in the early phase was higher in the proself group. These results appear to be due to the earlier establishment of predictions of the complex state transitions in the task. This can be explained if the proself group used model-based learning more intensively than the prosocial group, since the model-based process involves learning the probabilistic structure of the environment. In fact, this conjecture was confirmed by the results of simulations using HYBRID models with model-based and model-free parameters. Reward gains were greater for the proself group in the second block (approximately 60–80 trials) as well as in the first block, possibly due to the relatively small *w* and *β* in the prosocial group in the second block. With regard to the difference in learning rates between proselfs and prosocials, a previous study using the repeated prisoner's dilemma reported that the slope of the learning rate was steeper for proselfs^48^. However, it is not clear whether this difference in learning speed is due to the difference in the learning mechanisms, since the study did not use reinforcement learning model analysis. More generally, there is a finding that proselfs have faster reaction times than prosocials in many conditions^49^ and that participants with stronger model-based dependence have faster processing speeds than those with weaker ones^50^. It is possible that the differences in the behavioral outcomes found in this study are partially due to these basic differences in information processing.

The SVO slider measure we used in this study quantified the social preferences of the participants based on their choices of money allocation to the self and others. Proselfs focus on money to the self and disregard it to others. In our probabilistic state-transition task, the participants received additional money depending on their choices. Therefore, it is possible that the proselfs were more strongly motivated than the prosocials, or that proselfs placed a higher value on the rewards they received. While differences in motivation could be manifested in the overall reaction time, there was no significant difference in reaction time between the proself and prosocial groups in the first and second choices. In a previous study using a two-armed bandit task, the parameters representing the learning rate (*α*) and exploration (*β*) in the Q-learning model differed significantly depending on the presence or absence of rewards^51^. This result suggests that differences in motivation can be reflected in the learning rate and exploration tendency. However, in our study, there was no significant difference in learning rate and exploration tendency between the proself and prosocial groups, and the only difference was in the model-based dependence (*w*). It is difficult to completely rule out the possibility that the difference in the weight was affected by differences in motivation, but there is nothing in our results that positively supports this interpretation.

In recent years, many studies have used the “two-step task” to examine model-free and model-based behavior^3,11,12,14,17,40,52,53^. It has been suggested, however, that model-free strategies may masquerade as model-based when strategies beyond classical model-free reinforcement learning (i.e., strategies that learn about latent regularities in the task structure) are used for the two-step task^54^. If such a possibility arises in our task, it becomes difficult to argue with certainty that model-based strategies are being used there. However, as same study highlights^54^, such effects occur only with extensive training. Thus, for a limited number of trials like the current task (especially in its early stage), the problem of model-based strategies being masqueraded by model-free strategies is unlikely to occur.

We used a nonsocial decision-making task to investigate the relationship between social preferences and domain-general learning mechanisms. It is important to ask whether the findings here can be extended to social decision-making. Social interactions often involve the formation of (sometimes nested) internal models about others' minds ("theory of mind") and reasoning based on these models^55,56^. Previous studies using computer simulations have shown that social model-based strategies develop more deeply in competitive conditions; they remain relatively shallow in cooperative conditions^57,58^. These results may be consistent with our results that the proself (competitor) is more model-based dependent, while the prosocial (cooperator) is more model-free dependent.

The learning approach argues that the characteristics of social decision-making derive from those of the underlying learning mechanisms. This study examined the correspondence between individual differences in model-based/model-free dependence and individual difference in social preferences, and thus no definitive claims can be made regarding the causal relationship between the two. Another factor that may influence individual differences in SVO is environmental heterogeneity. Previous studies have shown that prosociality is enhanced in environments that are rewarding for cooperative behavior^59,60^. Note that the effect was found to be greater for participants who were more intuition-dependent, suggesting the influence of difference between model-based/model-free learning. Our study was conducted with university students in one country and did not examine or control for environmental heterogeneity. Future intercultural studies will be needed in this regard.

Since this experiment was conducted as an online experiment, the measurement of the reaction time may not be accurate. In a study that measured delays in reaction time in various online experiment platforms, reaction time delays in jsPsych, which was the platform used in this study, were not particularly slow on average or did not have greater variance than other platforms^61^. Still, systematic errors of up to 30 ms have been shown to occur, depending on the computer’s operating system and the browser used. Since we did not control for the experimental environment of the participants, we cannot deny the possibility that these errors may have affected the results to some extent.

The balance between model-based and model-free learning has been implicated in many psychiatric disorders and problematic behaviors. For example, it has been noted that the malfunction of model-based learning is associated with psychiatric disorders such as obsessive–compulsive disorder and schizophrenia, as well as problematic behavioral tendencies such as trait impulsivity^40,52,62^. In addition, dopamine replacement medication in healthy subjects and patients with Parkinson's disease has been shown to restore model-based learning^53,63^. This study has the potential to contribute to the understanding of social disorders in these psychopathologies by clarifying the relationship between reinforcement learning systems and prosociality.

In conclusion, this study revealed that, in a behavioral task involving stochastic state transitions, proselfs made decisions with a stronger influence of the model-based system and prosocials with that of the model-free system. The elucidation of these relationships is expected to lead to new insights into the mechanisms of prosociality and its inter- and intra-individual variations, as well as its evolutionary and developmental origins, from the perspective of domain-general learning mechanisms.

## Methods

### Participants

A total of 184 undergraduate students from Nagoya University and Aoyama Gakuin University (gender: 92 females, 1 no-answer; age: 18–36, 21.20 ± 1.67 SD) participated in our experiment online. All experimental protocols were approved by the ethics committee of the Brain Science Institute, Tamagawa University, according to the requirements of the Declaration of Helsinki, and were carried out in accordance with approved guidelines. Each participant signed an informed consent form to confirm his or her agreement to participate. One participant was excluded from the analysis because of missing personal information and SVO data.

### Behavioral task

The participants performed a sequential two-choice Markov decision task (as used in ref.^4^). In this task, each participant made two sequential choices (left or right key press) in the first and second stages. A monetary reward was probabilistically given, depending on the choice in the second stage. Each state was associated with a different geometric figure. The figure for the first stage were fixed, but the four figures used for the second stage (states L1, L2, R1, and R2) were randomized for each participant. In each stage, the stimulus was presented for 2 s, during which the participant had to press the left or right key on the keyboard. If the participant could not press either key within 2 s, the same trial was repeated after the warning message. The layout of the state transitions was a two-layer tree structure (Fig. 1a). Depending on the first left or right choice, the participant would transition to one of two possible intermediate states (state L1 or L2 for the left choice, state R1 or R2 for the right choice) and which one was determined probabilistically (common transition with 70% vs. rare transition with 30%). In the second stage, the left/right choice resulted in a 70% or 30% probability of moving to a state with a reward of either 0, 10, or 25 yen. Each reward amount was associated with three geometric figures. The relationship between each figure and the amount of reward was fixed throughout the experimental session. Reward amounts were readjusted to 0, 0.4, and 1 in the model analysis. Each stage was preceded by a fixation point ( +) for a randomly sampled time according to a uniform distribution of 2–4 s. An inter-trial-interval of 3–5 s was also inserted. Participants were first instructed on the basic task flow, and then given five demonstration trials for them to understand the task structure. In the demonstration trials, a participant did not make choices but was required to observe the result of the choices made automatically. The outcomes of the demonstration trials were not included in the participants’ rewards. Subsequently, 200 trials of the main task were conducted, and the participants made their choices by pressing keys. The behavioral task was developed using jsPsych version 6.20^64^, and the experiment was browser-based and conducted online using each participant’s computer.

### The SVO measure

The SVO measurement of participants’ social preferences was performed online using the SVO Slider Measure^20^. Participants were asked to assume an anonymous partner and make allocation choices without any real incentives. We used six and nine questions as the primary and secondary slider items, respectively, but only the results of the primary slider items were used to estimate SVO scores in the analysis.

### Participant payoff

Participants were paid a show-up fee of 1000 yen and the sum of the reward amounts earned in the 200 trials. Payment was made by bank transfer from the university to the participant's account. The entire experiment took approximately 40 min to an hour.

### Behavioral analysis

Offline analysis was carried out using custom-made MATLAB programs (R2021b, MathWorks, MA, USA). GLMEs were used with gender as a fixed effect and age as a random effect to examine the effects of gender on SVO score, and vice versa for the effect of age, using the *glmfit* function of MATLAB. Based on the SVO scores, we classified the participants into two groups: a prosocial group, with a score of 37.48° (indicating inequity-aversion) or higher, and a prosocial group, with a score of 7.82° (indicating complete individualism) or lower. To compare the reward amounts in the two groups, we calculated the moving average of the reward amount every 20 trials in one trial step for each participant using the *movemean* function of MATLAB. With this function, if there are not enough elements to fill the window at both ends, the window size is automatically truncated. If the window is truncated, the average value is taken only from the elements that fill the window. We then performed a Wilcoxon’s rank-sum test with the data collected for each group. Reaction time (at the first stage: RT1 and the second stage: RT2) was measured as the time from the stimulus onset to the key press, which was obtained from the output data of jsPsych. The RTs of the first trial of the session were excluded from the analysis. For RT1, RT2 after common transitions, and RT2 after rare transitions, a repeated measure two-way ANOVA was conducted using social preferences (proself/prosocial) and blocks (five blocks of 40 trials each) as factors. For RT2 in the prosocial and proself groups, a two-way ANOVA was conducted using blocks and transition probabilities (common/rare transitions) as factors. For RT1, a GLME was used with trial number and social preference as fixed effects; participant ID, gender, and age as random effects. For RT2, a GLME was used with trial number, social preference, and transition probabilities as fixed effects; participant ID, gender, and age as random effects. For post-hoc analysis, comparisons between blocks in each group were carried out using a paired samples *t*-test, and comparisons between groups per block were carried out using an unpaired two-sample *t*-test. As complementary analysis, the same statistical comparisons were applied to the prosocial and proself groups classified by the conventional criterion of more (prosocial) or less (proself) than 22.45° in SVO score.

### Computational learning models

We implemented three learning models in this study following a previous study^4^. Each model was assumed to learn about state-action values based on the participant's experience of actions, state transitions, and rewards. We divided the total 200 trials into blocks of 40 trials and calculated the estimates of the state-action values for each block. The estimates of state-action values and parameters at the end of each block were carried over as initial values to the estimation in the next block.

### SARSA learner

As a pure model-free learner, we used the SARSA model, a classical reinforcement-learning model. The name SARSA is derived from a set of symbols used in the formulation of the model, (*s, a, r, s’, a’*), where *s* is the current state, *s’* is the next state, *a* is the current action, *a’* is the next action, and *r* is the reward obtained when the transition to the next state is made. The learner updates the state-action value *Q*_*SARSA*_(*s*,*a*) corresponding to action *a* in state *s* according to the following formula:

$${Q}_{SARSA}\left(\mathrm{s},\mathrm{a}\right)\leftarrow {Q}_{SARSA}\left(\mathrm{s},\mathrm{a}\right)+\mathrm{\alpha }{\delta }_{RPE},$$

$${\delta }_{RPE}=r\left({s}^{{\prime}}\right)+ \gamma \cdot {Q}_{SARSA}\left({s}^{{\prime}},{a}^{{\prime}}\right)-{Q}_{SARSA}(s,a)$$

Here, *δ*_*RPE*_ is the reward prediction error, which is calculated for each stage and used to update the state action value. *α* is a free parameter that controls the learning rate, and *γ* is the time discount factor. Since the task in this study did not include choices that are rewarded at different times, *γ* was fixed at 1. State transition probabilities were not considered in this model.

### FORWARD learner

As a pure model-based learner, we used the FORWARD model, which involves the calculation of state prediction errors. The estimated probability of transition to state *s’* when action *a* is selected in state *s* is denoted by *T*(*s*, *a*, *s’*). In FORWARD model-based reinforcement learning, this estimate is used to calculate state-action values. The estimated state transition probability to transited state *s’* is updated according to the following formula:

$$T\left(s,a,{s}^{{\prime}}\right)\leftarrow T\left(s,a,{s}^{{\prime}}\right)+ \eta {\delta }_{SPE},$$

$${\delta }_{SPE}=1-T\left(s,a,{s}^{{\prime}}\right),$$

*δ*_*SPE*_ is the state prediction error, and *η* is a free parameter that controls the learning rate.

For state *S'﻿'*, where the agent did not transition to, the estimated state transition probability is updated as follows:

$$T\left(s,a,s{^{\prime}}{^{\prime}}\right)\leftarrow \left(1- \eta \right)T(a,s,a{^{\prime}}{^{\prime}})$$

Using the estimated state transition probabilities, the state action value *Q*_*FWD*_(*s*,*a*), corresponding to action *a* in state *s*, is updated as follows:

$${Q}_{FWD}\left(\mathrm{s},\mathrm{a}\right)= \sum_{S{^{\prime}}}T\left(s,a,{s}^{{\prime}}\right)\cdot \left[E[r\left({s}^{{\prime}}\right)] +\underset{\mathit{a{^{\prime}}}}{\mathrm{max}}{Q}_{FWD}({s}^{{\prime}},{a}^{{\prime}})\right]$$

*E*[*r*(*s’*)] is the expected value of the reward obtained when transitioning to state *s’*. Thus, in the FORWARD learner model, the state-action value is calculated based on the state transition probability and the reward obtained in each state.

### HYBRID learner

We used a third learner model, the HYBRID learner, which combines the estimates of state-action values from both SARSA and FORWARD learners with a certain weight to calculate a new estimate. In a previous study^4^, weight was given as an exponential function that decayed trial by trial. Since we conducted our estimation in blocks of 40 trials, weight was represented as a scalar *w* fixed within each block. *w* = 0 represents pure model-free, and *w* = 1 represents pure model-based. The state-action value *Q*_*HYB*_(*s*,*a*) of the HYBRID learner is calculated as follows:

$${Q}_{HYB}\left(s,a\right)=w\cdot {Q}_{FWD}\left(s,a\right)+(1-w)\cdot {Q}_{SARSA}(s,a)$$

### Action selection

In each model, the agent is assumed to generate actions stochastically according to a softmax function in each state of each trial. The choice probability *P*(*s*,*a*) of action *a* in state *s* is calculated using the following formula:

$$P\left(s,a\right)= \frac{\mathrm{exp}(\beta \cdot Q\left(s,a\right))}{\sum_{a{^{\prime}}}\mathrm{exp}(\beta \cdot Q\left(s,a{^{\prime}}\right))}$$

Here, *Q* is the action-state value (either *Q*_*SARSA*_, *Q*_*FWD*_, or *Q*_*HYB*_) corresponding to the model under consideration. *β* is a free parameter, called the “inverse temperature.” A larger *β* indicates that the choice of an action is more sensitive to differences in opposing action values. In other words, the smaller the *β*, the more exploratory the choice will be under the same action-state values.

### Parameter estimation

We fitted the free parameters (*α*, *η*, *β*, *w*) of each model to the behavioral data of each participant using negative log-likelihood to make statistical comparisons between the prosocial and proself groups. To eliminate extreme value estimation that tends to occur in individual-level analysis, we introduced a maximum a posteriori (MAP) estimation. In a MAP estimation, we estimated parameters that minimize the negative log posterior probability density, which is the sum of the negative log likelihood and the negative log prior probability density. Specifically, following a previous study^65^, we used the beta distribution (beta[2, 2]) as the prior distribution of the learning rate *α* and *η*, and the gamma distribution (gamma[2, 3]) as the prior distribution of the inverse temperature *β* as the regularization term for the log likelihood. The *fmincon* function of MATLAB was used with the constraints that 0 < *α*, *η*, *w* < 1 and 0 < *β* < 10 when performing the MAP estimation. As described above, 200 trials were divided into blocks of 40 trials, and parameter estimation was performed for each block. The estimated state-action values and free parameters in the last trial of each block were carried over as initial values for the next block. To check the validity of the estimates of the individual-level analysis, we also conducted a population-level fixed-effects analysis for the proself and prosocial groups with the same constraints. In the fixed-effects analysis, the free parameters were set to the same values among participants within each group.

To perform between-group comparisons of the estimated parameters, we divided each estimate into proself and prosocial groups and conducted a repeated measures two-way ANOVA with social preference and block as factors. In addition, GLMEs were used with block and social preference as fixed effects; participant ID, gender, and age as random effects. Comparisons were then made between blocks in the same group and between groups in each block using the Wilcoxon rank-sum test as a post-hoc analysis. To create correlation matrices for each group and each block, correlations between parameters were calculated using Spearman's rank correlation coefficients.

### Simulating model learners

We iteratively simulated the behavior of model-based and model-free learners using the HYBRID model. In this simulation, the values of the learning rate and inverse temperature were set to the values estimated in the fixed-effects analysis in a previous study (*α*, *η* = 0.2, *β* = 5.0)^4^ which used the same task and the same model as in this study. The value of weight *w* was set to 1 for the first 40 trials and 0.5 for the subsequent trials in the simulation of the model-based learner. Similarly, the weight was set to 0 for the first 40 trials and 0.5 for subsequent trials in the simulation of the model-free learner. We ran 10,000 repetitions of model-based and model-free simulations of the sequential two-choice Markov decision task, and pooled the outcomes obtained in each simulation cycle.

The difference between the estimated state action values of the left and right choices in the first stage is denoted as *Q*(1,R)–*Q*(1,L). This difference should approach to the true value as the trial progresses. To investigate the learning speed in each learner based on this difference, we fitted *Q*(1,R)–*Q*(1,L) in each simulation cycle using a sigmoid function passing through the origin as follows:

$$\mathrm{sigfunc}= \frac{a}{1+\mathrm{exp}(-b*x)}-\frac{a}{2}$$

*a* represents the value of the plateau and *b* represents the slope (steepness of the curve). The magnitude of *b* corresponds to the learning speed. In estimating *a* and *b*, we used the *nlinfit* function of MATLAB, which was used to fit the nonlinear regression model. To make statistical comparisons of simulated values between different learners, we used a trial-by-trial unpaired two-sample *t*-test for reward amount and the Wilcoxon rank-sum test for slope.

We also ran similar simulations to obtain recovery statistics using parameters estimated from the behavior of the proself and prosocial groups. The parameter values obtained using fixed effects analysis were used here.

## Supplementary Information

Supplementary Figures.

## Data Availability

All data analyzed in the current study and the MATLAB codes used to run our statistical analyses are available in an Open Science Framework repository (https://osf.io/hvjce/).
