# Supplementary material for: Proselfs depend more on model-based than model-free learning in a non-social probabilistic state-transition task
Source: Sci Rep. 2023 Jan 25;13:1419. doi: 10.1038/s41598-023-27609-0 (PMC9876908; doi:10.1038/s41598-023-27609-0)
Supplement: Supplementary file 1 — Supplementary Figures. [file 41598_2023_27609_MOESM1_ESM.docx]

**Supplementary Information for**

Proselfs depend more on model-based than model-free learning in a non-social probabilistic state-transition task

Mineki Oguchi^1†^, Yang Li^1,2†^, Yoshie Matsumoto^1,3^, Toko Kiyonari^4^, Kazuhiko Yamamoto^5^, Shigeki Sugiura^5^, and Masamichi Sakagami^1*^

1. Brain Science Institute, Tamagawa University, Tokyo, Japan

2. Graduate School of Informatics, Nagoya University, Nagoya, Japan

3. Department of Psychology, Faculty of Human Sciences, Seinan Gakuin University, Fukuoka, Japan

4. School of Social Informatics, Aoyama Gakuin University, Tokyo, Japan.

5. Genesis Research Institute, Aichi, Japan.

^†^ These authors contributed equally to this work.

* Corresponding author: Masamichi Sakagami

[Address] Brain science institute, Tamagawa University, 6-1-1, Tamagawagakuen, Machida, Tokyo, Japan

Phone: +81-42-739-8679

Fax: +81-42-739-7220

**Email:** [sakagami@lab.tamagawa.ac.jp](mailto:sakagami@lab.tamagawa.ac.jp)

**This PDF file includes:**

Figures S1 to S7


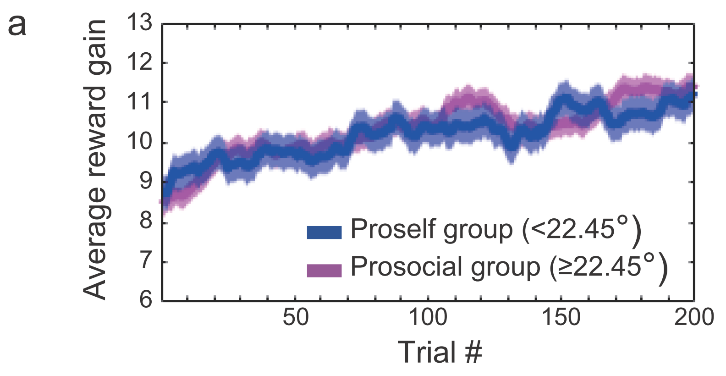


**Fig. S1.** Comparing reward gain between the proself and prosocial groups divided by SVO angle 22.45° (a), moving averages of the reward amount for the proself and prosocial groups; Blue indicates the proself group (SVO<22.45°). Magenta indicates the prosocial group (SVO≥22.45°).


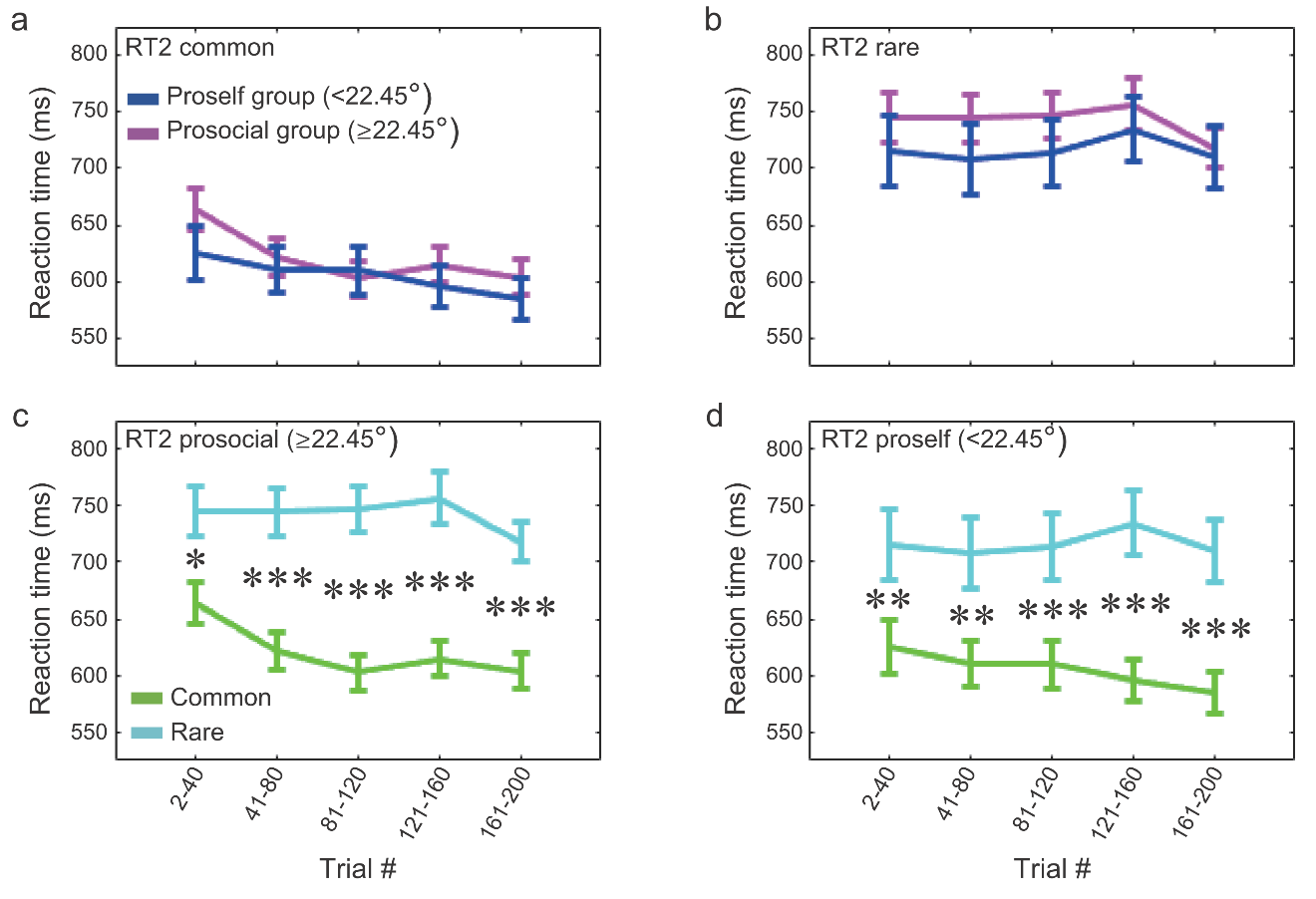


**Fig. S2.** Comparing reaction times between the proself and prosocial groups divided by SVO angle 22.45° (a), Averaged reaction times after common transitions per 40 trials at the second choice, divided into the proself (SVO<22.45°) and prosocial (SVO≥22.45°) groups. A two-way repeated measures ANOVA with social preference and block as factors showed a main effect of block (*F*[4,181] = 9.43, *p* = 1.93×10^-7^). Blue indicates the proself group. Magenta indicates the prosocial group. Error bar indicates SEM. (b), RTs at the second choice after rare transitions. A two-way repeated measures ANOVA showed an interaction (*F*[4,181] = 3.04, *p* = 0.017). There was no significant difference in either block comparing RT2 after common and rare transitions. (c), RTs at the second choice of the prosocial group divided into after common and rare transitions. A two-way repeated measures ANOVA with transition probability and block as factors showed main effects of transition probability (*F*[1,116] = 129.87, *p* = 1.21×10^-20^), block (*F*[9,116] = 30.38, *p* = 2.19×10^-47^), and their interaction (*F*[1,116] = 130.26, *p* = 1.10×10^-20^). Comparing RT2 after common and rare transitions block by block, there were significant differences from the first to the end blocks (Block1: *t*[232] = 2.21, *p* = 0.028, Block2: *t*[232] = 4.24, *p* = 2.06×10^-5^, Block3: *t*[232] = 5.08, *p* = 7.77×10^-7^, Block4: *t*[232] = 5.02, *p* = 1.03×10^-6^, Block5: *t*[232] = 4.14, *p* = 4.85×10^-5^, two-sample *t*-test). Green represents after common transitions, and cyan represents after rare transitions. (d) RTs at the second choice of the proself group. A two-way repeated measures ANOVA with transition probability and block as factors showed main effects of transition probability (*F*[1,65] = 104.69, *p* = 3.44×10^-15^), block (*F*[9,65] = 23.70, *p* = 1.23×10^-34^), and their interaction (*F*[1,65] = 94.08, *p* = 2.96×10^-14^). Comparing RT2 after common and rare transitions block by block, there were significant differences from the first to the end blocks (Block1: *t*[130] = 3.16, *p* = 0.002, Block2: *t*[130] = 3.04, *p* = 0.003, Block3: *t*[130] = 3.84, *p* = 1.91×10^-4^, Block4: *t*[130] = 4.29, *p* = 3.45×10^-5^, Block5: *t*[130] = 4.97, *p* = 2.02×10^-6^). *: *p* < 0.05, **: *p* < 0.01, ***: *p* < 0.001.


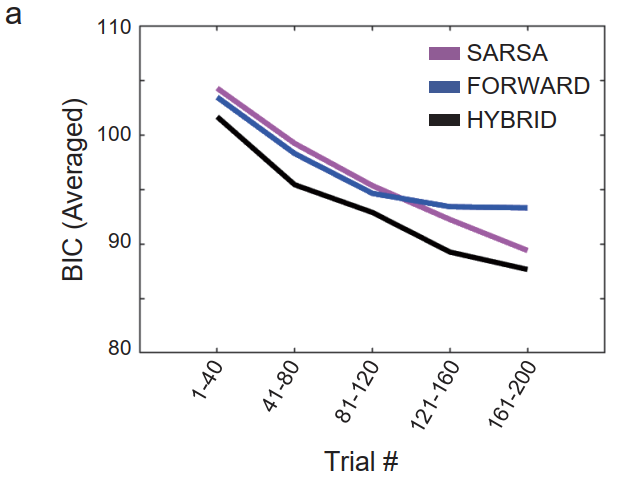


**Fig. S3**. Goodness-of-ﬁt comparison between models (a), Bayesian Information Criterion (BIC) computed for the model fitting of the model-free SARSA learner (magenta), model-based FORWARD learner (blue), and the HYBRID learner (black) in every 40 trials for all participants’ data. The vertical line indicates BIC scores averaged over participants.


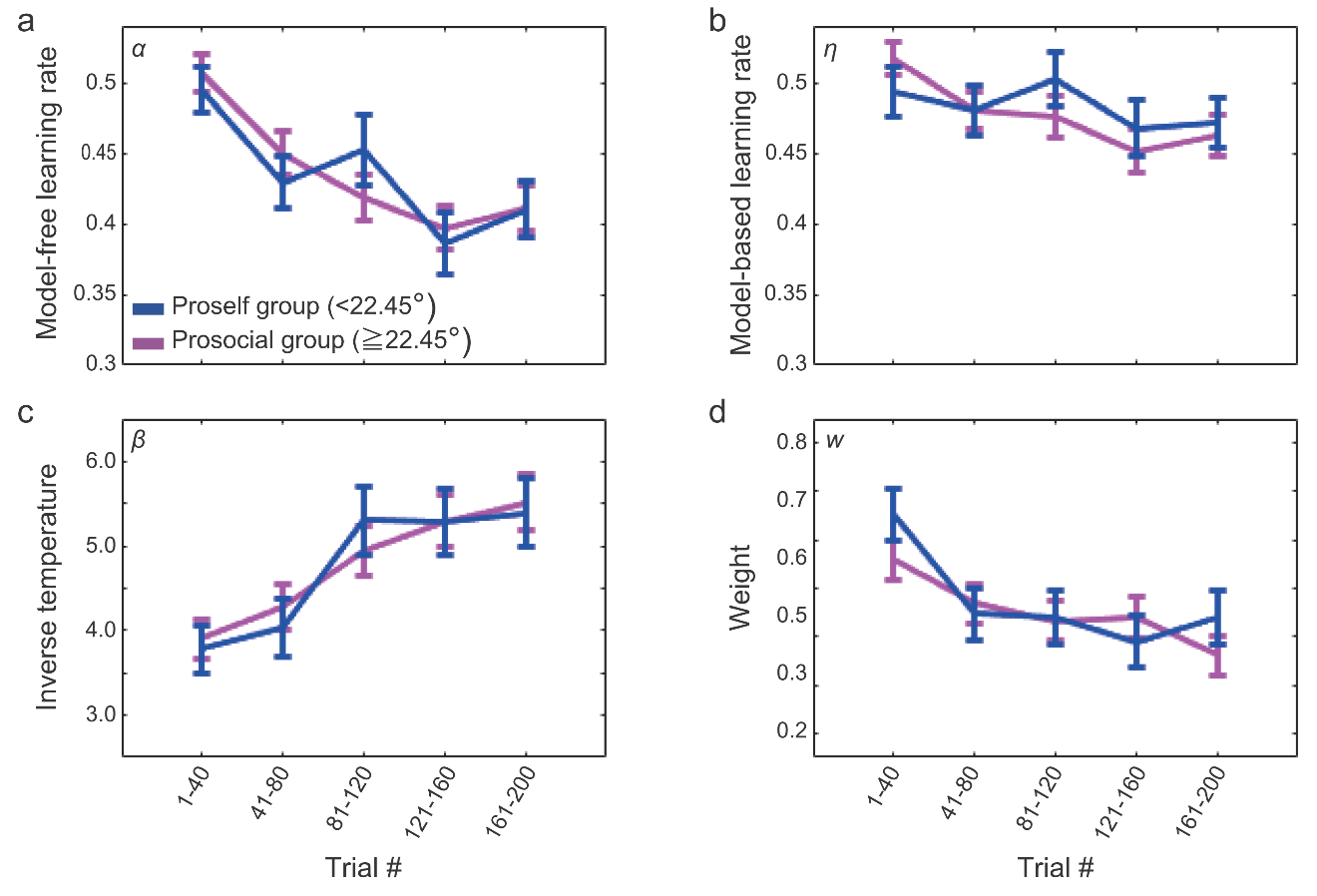
**Fig. S4.** Comparing estimated parameters between the proself and prosocial groups divided by SVO angle 22.45° (a), model-free learning rate (*α*) estimated by the model analysis, divided into the proself (SVO<22.45°) and prosocial (SVO≥22.45°) groups. A two-way repeated measures ANOVA, with social preference and block as factors, showed a main effect of block (*F*[4,181] = 10.83, *p* = 1.56×10^-8^). Blue indicates the proself group; magenta indicates the prosocial group. Error bar indicates SEM. (b), model-based learning rate (*η*). A main effect of block (*F*[4,181] = 2.66, *p* = 0.032) was found. (c), inverse temperature (*β*). A main effect of block (*F*[4,181] = 17.46, *p* = 1.08×10^-13^) was found. (d), Weight (*w*) representing model-based dependence. A main effect of block (*F*[4,181] = 6.19, *p* = 6.71×10^-5^) was found. The interaction was not significant for α, η, β, or w, even with the first 80 trials.


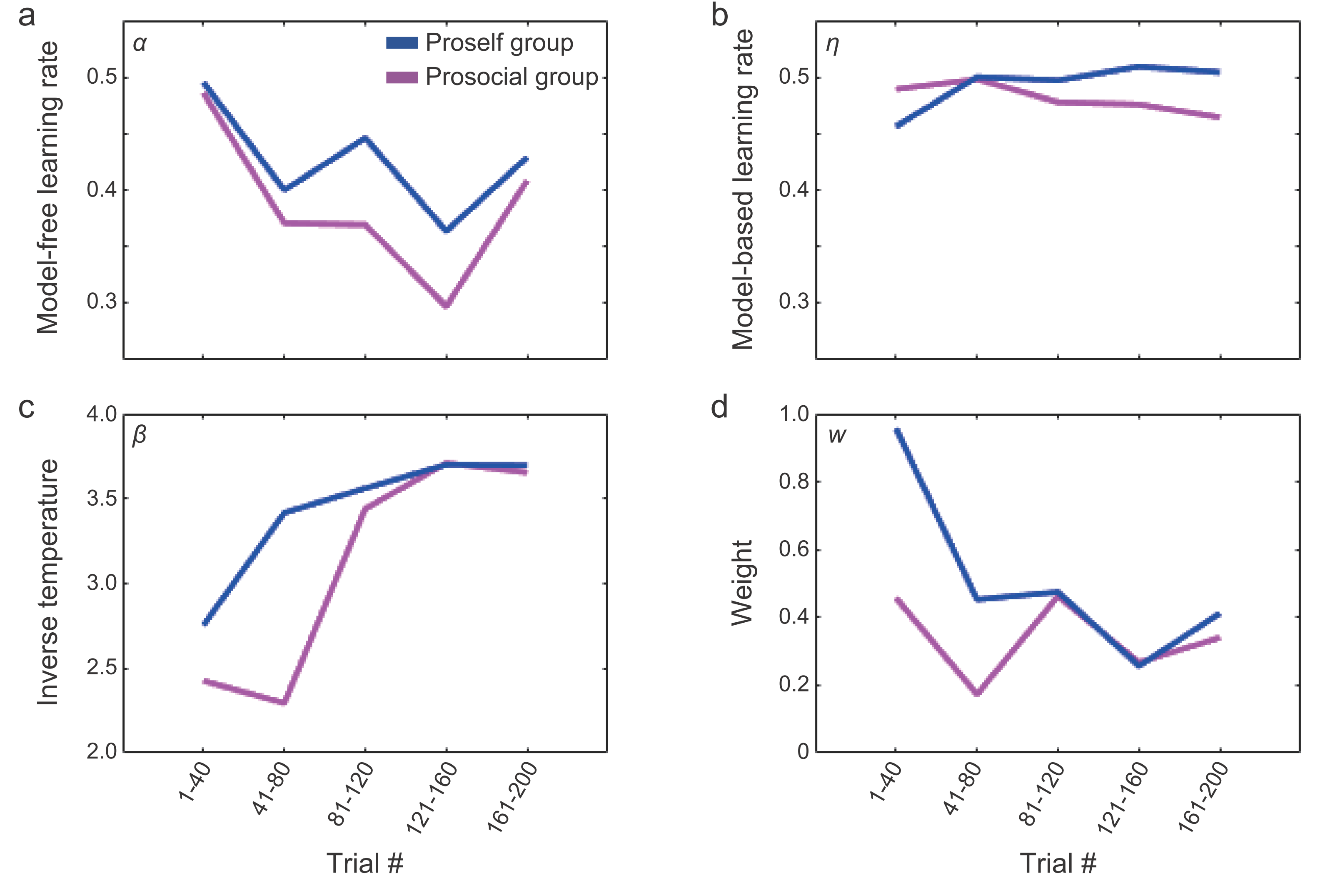


Fig. S5. Estimating model parameters with fixed-effect analysis (a), model-free learning rate (*α*) calculated using the fixed-effects analysis, divided into the proself group (blue) and prosocial group (magenta). (b), model-based learning rate (*η*). (c), inverse temperature (*β*). (d), weight (*w*) representing the model-based dependence.


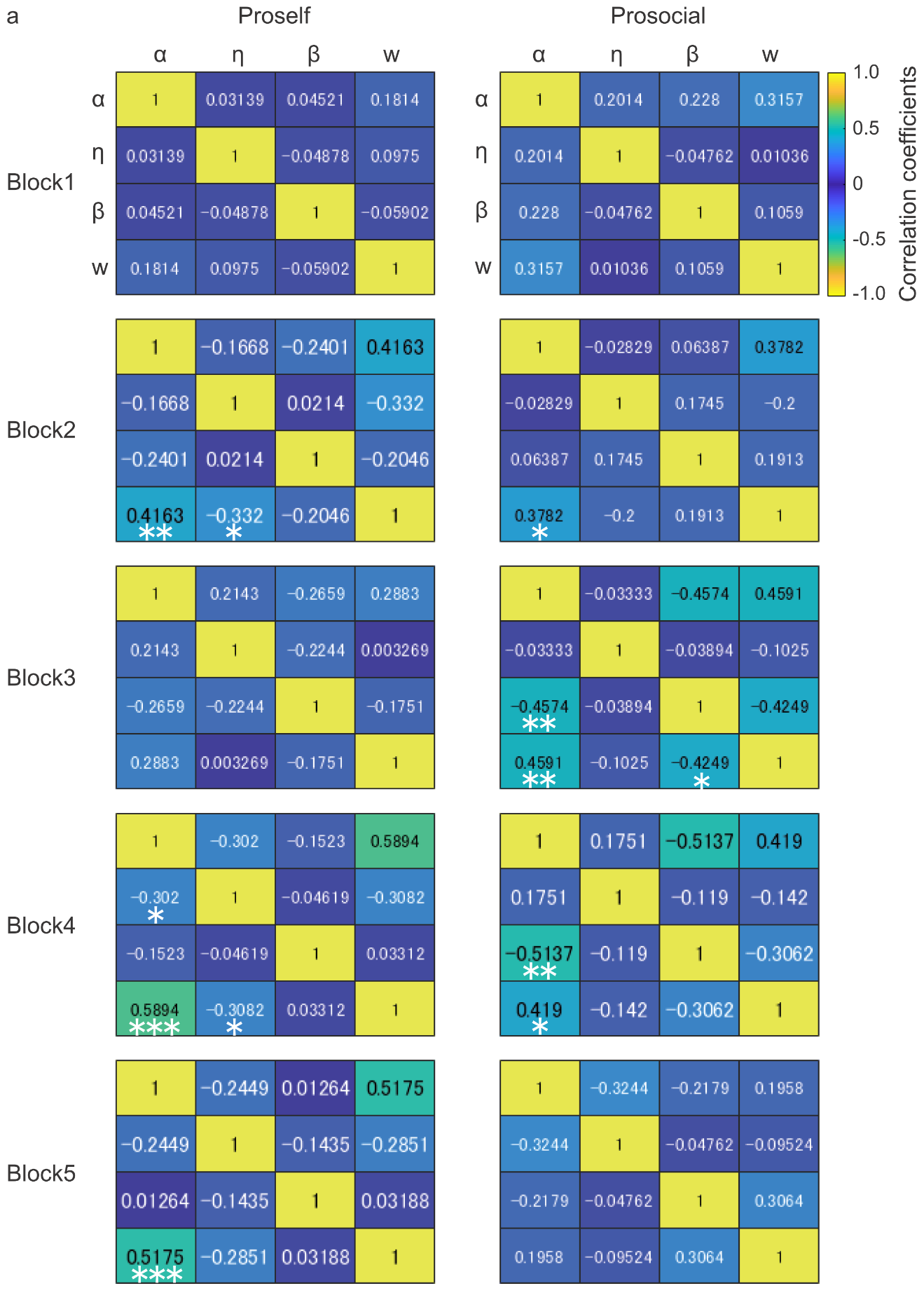


**Fig. S6.** Correlation matrices between the free parameter estimates in the HYBRID model using individual-level analysis. (a), The left column indicates the proself group, the right column indicates the prosocial group, and the rows indicate blocks. The numbers in the cells are Spearman's rank correlation coefficients. *: p < 0.05, **: p < 0.01, ***: p < 0.001.

**Fig S7.** Simulating the HYBRID model using the parameter estimates obtained by fixed effects analysis for the proself and prosocial groups (the proself and prosocial actors). (a), Difference between rewards earned by the proself and prosocial actors. * indicates p < 0.001. (b), state-action values at the first choice (Q[1,R] for the right choice, Q[1,L] for the left choice) estimated from simulations of the proself (blue) and prosocial (magenta) actors. (c), the histogram of the slope of the sigmoid function fitted to the difference of state action values in (b). The dotted lines represent the mean. The slope values were larger for the proself than for the prosocial actors (*z* = 7.00, *p* = 2.53×10^-12^, Wilcoxon rank-sum test)
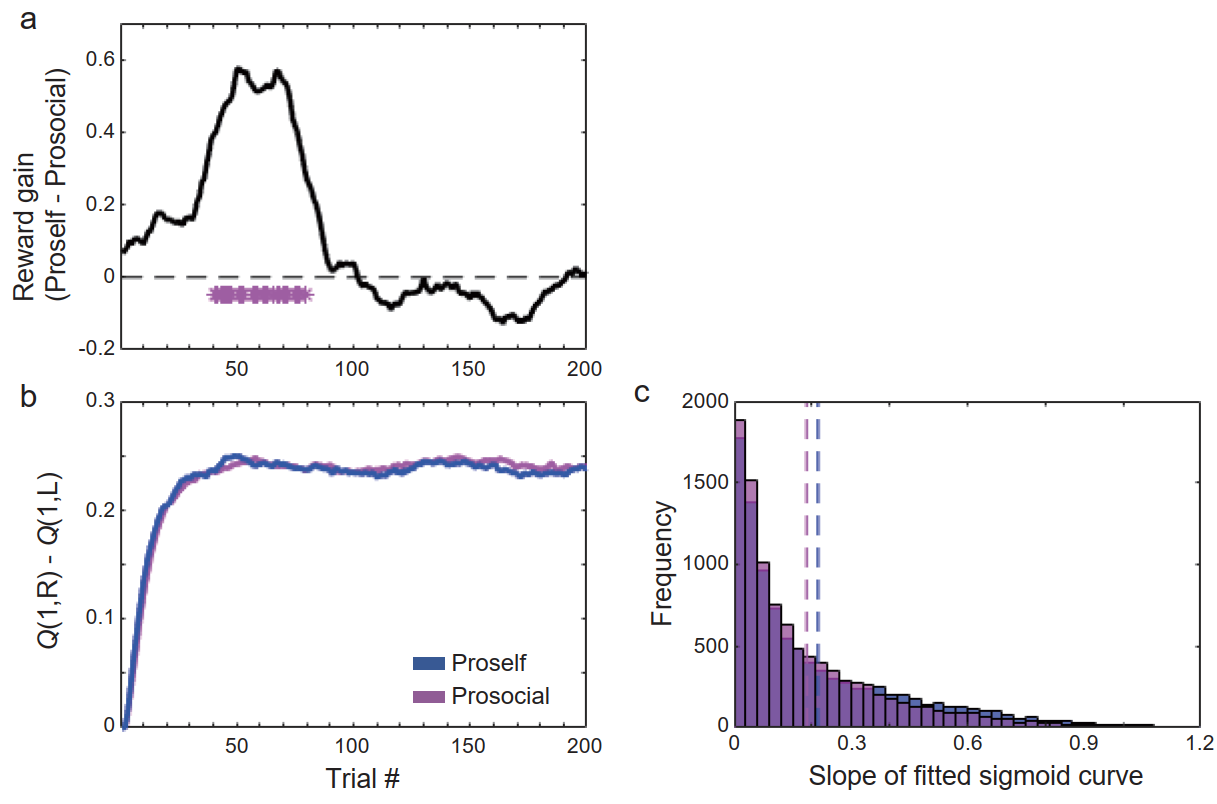
.
